# Use of Piranha Solution as An Alternative Route to Promote Bioactivation of PEEK Surface with Low Functionalization Times

**DOI:** 10.3390/molecules28010074

**Published:** 2022-12-22

**Authors:** Flavia Suzany Ferreira dos Santos, José Filipe Bacalhau Rodrigues, Milena Costa da Silva, Maria Eduarda Vasconcelos Barreto, Henrique Nunes da Silva, Suédina Maria de Lima Silva, Marcus Vinicius Lia Fook

**Affiliations:** Department of Materials Engineering, Federal University of Campina Grande, Campina Grande, PB 58429-900, Brazil

**Keywords:** PEEK, surface bioactivation, sulfuric acid, piranha solution, sulfonic acid functional group

## Abstract

This study aimed to achieve bioactivity on the PEEK surface using piranha solution through a lower functionalization time. For this purpose, the functionalization occurred with piranha solution and 98% sulfuric acid in the proportions of 1:2, 1:1, and 2:1 at periods of 30, 60, and 90 s. The samples treated for longer times at higher concentrations registered the characteristic spectroscopy band associated with sulfonation. Additionally, both chemical treatments allowed the opening of the aromatic ring, increasing the number of functional groups available and making the surface more hydrophilic. The piranha solution treatments with higher concentrations and longer times promoted greater heterogeneity in the surface pores, which affected the roughness of untreated PEEK. Furthermore, the treatments induced calcium deposition on the surface during immersion in SBF fluid. In conclusion, the proposed chemical modifications using sulfuric acid SPEEK 90 and, especially, the piranha solution PEEK-PS 2:1-90, were demonstrated to be promising in promoting the rapid bioactivation of PEEK-based implants.

## 1. Introduction

Poly (ether-ether-ketone)—PEEK—has stood out in its use in orthopedic implants due to its characteristics of biocompatibility and mechanical and chemical resistance [1]. Moreover, it has mechanical properties, such as elastic modulus and tensile strength, closer to those of human bones when compared to metal implants [2]. The PEEK polymer is known as an alternative biomaterial for implantable metallic materials. However, it is biologically inert, and this technical feature prevents the occurrence of interactions between an implant and bone tissues. Osseointegration is essential for bone regeneration, i.e., without this condition, the implants may loosen or migrate, causing pain to the patient, deformity, or deficiency [3,4].

Several approaches have been focused on overcoming the inert character of PEEK using physical or chemical modifications to improve the bone-implant interface [5,6,7,8,9,10]. The physical treatments commonly used are plasma modifications and accelerated neutral atom beams. In plasma treatment, some metastable species induce polymer functionalization, generating free radicals in the chains. For example, the insertion of oxygen-containing polar groups on the PEEK surface increases hydrophilicity and generates greater cell adhesion [11,12]. In addition, there is evidence that the treatment reduces the adhesion of bacteria to the polymer surface [13].

Regarding chemical processes, only wet modification or sulfonation treatment can change the surface chemical structure of the material, creating a new physicochemical environment favorable to cellular response [3,14]. Furthermore, PEEK can be applied as a coating to provide bioactive effects on some materials through various methods, including the cold spray technique, radiofrequency, sputtering, ionic plasma deposition, and electron beam deposition, among others. Surface treatment alone or in combination with surface coating can significantly improve PEEK bioactivity [3].

Sulfonation has become a promising alternative for the chemical modification of the PEEK surface. Concentrated sulfuric acid (98%) is selected as the sulfonating agent because the reaction is simple. Sulfuric acid is a reagent known to produce polymers free from degradation and crosslinking reactions [15,16]. Therefore, it is very effective to introduce the sulfonic acid functionality into the polymer chain to make it hydrophilic and increase its bioactivity [17].

Different approaches regarding the chemical modification of PEEK with sulfuric acid have been reported in the literature. Almasi et al. [18] studied many immersion times of PEEK in sulfuric acid and its effects on the polymer chain. Wang et al. [19] evaluated the hydrophilicity and morphology of PEEK under different sulfonation conditions. They observed an increase in the hydrophilic character of the modified surface in comparison to the unmodified one. Montero et al. [20] analyzed the antimicrobial behavior of SPEEK (sulfonated PEEK) membranes against *Streptococcus mutans* and *Enterococcus faecalis* colonies and reported that the sulfonation process affected the growth of SPEEK biofilm, which reveals a potential modification of PEEK aiming at antimicrobial activity. Zhao et al. [21] observed that the surface modification sulfonation technique improved the adhesion and cell proliferation of PEEK in addition to inducing apatite formation on the modified surface.

Another chemical modification technique that has been studied is the use of a solution of sulfuric acid with hydrogen peroxide (H_2_O_2_), named “piranha solution”, which is a powerful corrosive and oxidizing agent. Its reported effects include increases in hydrophilicity and surface energy due to the increased number of functional groups available as a result of the synergistic action of its constituents [5,22,23,24,25,26,27].

Both concentrated sulfuric acid and piranha solution change the chemical characteristics of the PEEK surface and therefore promote bioactivity. Hence, chemical modification can act synergistically on the surface, producing strong bonds between PEEK and the substrate, promoting bioactivity, and thus decreasing the time of osseointegration between bone and PEEK [5,18,23,26].

Some studies using chemical modification on the PEEK surface by sulfuric acid were previously carried out. However, this route associated with piranha solution as a chemical modifying agent with low functionalization times and different concentrations is not well-known in the literature. Thus, this work aimed to achieve bioactivity on the PEEK surface using piranha solution through a lower functionalization time. Moreover, this work evaluates the influence of the treatment on the physicochemical, morphological, and biological properties of the modified PEEK surface.

## 2. Materials and Methods

### 2.1. Sample Preparation

**Figure 1:**
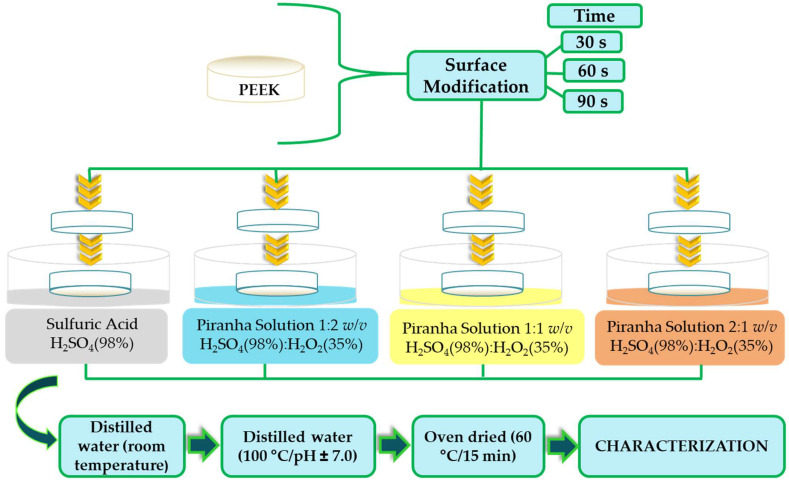
Schematic diagram of the methods used for sample preparation.

First, PEEK rods (TECAPEEK, Classix White Victrex^®^) for medical-technical applications (Ensinger, São Leopoldo, Brazil) were sectioned using a horizontal mechanical Imor^®^ lathe (São Paulo, Brazil), obtaining cylindrical specimens (20.0 mm diameter and 8 mm thick). Then, a metallographic PLO_2_ E Teclago^®^ polishing machine (São Paulo, Brazil) was utilized to wet-grind the sample with 600-, 1200-, and 2000-grit aluminum carbide sandpaper (Deerfos Abrasivos, Maringá, Brazil). Wet-ground specimens were ultrasonically cleaned in a distilled water (electric conductivity 0.8 µS/cm) bath for 10 min (Ultra Cleaner 1440 plus Unique, São Paulo, Brazil) to remove the remaining abrasive particles on the surface. Subsequently, the samples were oven-dried at 60 °C for 20 min and afterward were etched in (i) 98% sulfuric acid P.A. (Dinâmica Química Contemporânea, São Paulo, Brazil) and (ii) 98% sulfuric acid and 35% hydrogen peroxide P.A. (Neon, São Paulo, Brazil), named piranha solution (H_2_SO_4_:H_2_O_2_ = 1:2, 1:1 and 2:1 *w*/*v*) for 30, 60, and 90 s (Table 1). The specimens were rinsed repeatedly with distilled water at room temperature and subsequently washed with distilled water at 100 °C until the complete removal of sulfuric acid (pH ≅ 7.0), ensuring no residual acid in the sample, as described previously [20,21]. The pH measurements were conducted with a Q400A pH meter (QUIMIS, São Paulo, Brazil). Figure 1 shows a schematic diagram of the method used for sample preparation.

### 2.2. Characterization

#### 2.2.1. Infrared Spectroscopy by Fourier Transform (FTIR)

FTIR studies were performed to detect any chemical bonding between PEEK, SPEEK, and PEEK-PS using a Perkin Elmer Spectrophotometer Spectrum 400 (Waltham, MA, USA) equipped with an attenuated total reflectance (ATR, ZnSe Crystal) accessory in the absorbance mode. The FTIR analysis applied a scanning range in the average infrared region of 4000 to 650 cm^−1^, working with 32 scans and with a resolution of 4 cm^−1^.

#### 2.2.2. Surface Wettability

The wettability of PEEK surfaces was investigated through the contact angle of the water (θ) measurement by the sessile drop method. A contact angle goniometer (KRÜSS GmbH, Hamburg, Germany) was employed to determine the θ values. An ultrapure water drop was placed on the PEEK sample surfaces (2.0 µL) using a precision syringe with a needle diameter of 0.75 mm [27]. A video camera captured images of the water droplets, taken for 5 s, and their profiles were numerically solved and fitted to the Laplace-Young equation. The mean values of θ were collected from five different points in each sample. Untreated PEEK was considered as the control surface.

#### 2.2.3. Scanning Electron Microscopy (SEM)

SEM was used for the evaluation of the surface morphology of the specimens, after surface chemical modification, with a Hitachi model TM-1000 (Chiyoda, Tokyo, Japan) electronic scanning electron microscope with a maximum magnification of x, a depth of focus of 1 mm, a resolution of 30 nm, 15 kV, low vacuum and varied pressure (1 to 270 Pa), and without metallic coating. For the application of this technique, increases of 3000× and 6000× were applied.

#### 2.2.4. Surface Roughness Measurement

The surface roughness average (Ra) is the most commonly used parameter to evaluate surface properties. In this study, the software Gwyddion 2.45 (Okružní, Tchéquia) was used to determine the surface rugosity. Additionally, it provided a systematic characterization of the pore surface roughness and the pore structure in two and three dimensions. The data were obtained with the SEM micrographs and expressed from the root mean square (Rms) and roughness average (Ra) perspectives.

#### 2.2.5. In Vitro Bioactivity

The in vitro bioactivity of the untreated PEEK and the modified PEEK was evaluated by soaking them in a simulated body fluid (SBF), which was prepared according to the method of Kokubo and Takadama [28]. Each sample was immersed in 40 mL of SBF at 37 °C for 14 and 21 days in an orbital shaker, refreshing the SBF every seven days. After being removed from the SBF, they were gently rinsed with distilled water, dried at room temperature, and finally inspected by SEM.

#### 2.2.6. Statistical Analysis

Infrared spectroscopy (FTIR): Student’s t-test was applied on the absorbance averages of the O–H and S=O bands to identify the significant differences between the surface treatment methods. The data were evaluated using the Action Stat version 3.6.331.450 software (São Carlos, SP, Brazil). All samples were analyzed in three different regions to obtain the means corresponding to the values of the O–H and S=O absorbance. A statistical significance level of *p*-value < 0.05 was considered for all tests.

Surface roughness measurement: To evaluate the differences between the surface chemical treatment methods on the Rms and Ra values, Student’s t-test was applied. The Rms and Ra values were collected using the software Gwyddion 2.45 applied to the micrography images. The Rms and Ra data were obtained from three different regions (*n* = 3) of the samples. The data were evaluated using the Action Stat version 3.6.331.450 software (São Carlos, SP, Brazil). The significance level was 95%, and significant differences between means were adopted for *p*-value < 0.05. For a *p*-value > 0.05, no significant difference was observed between samples.

For surface wettability, contact angle data were obtained from five different points in each sample (*n* = 5). The adopted significance level was 95%. For *p*-value < 0.05 the difference between means was considered significant.

## 3. Results and Discussion

### 3.1. Infrared Spectroscopy by Fourier Transform (FTIR)

**Figure 2:**
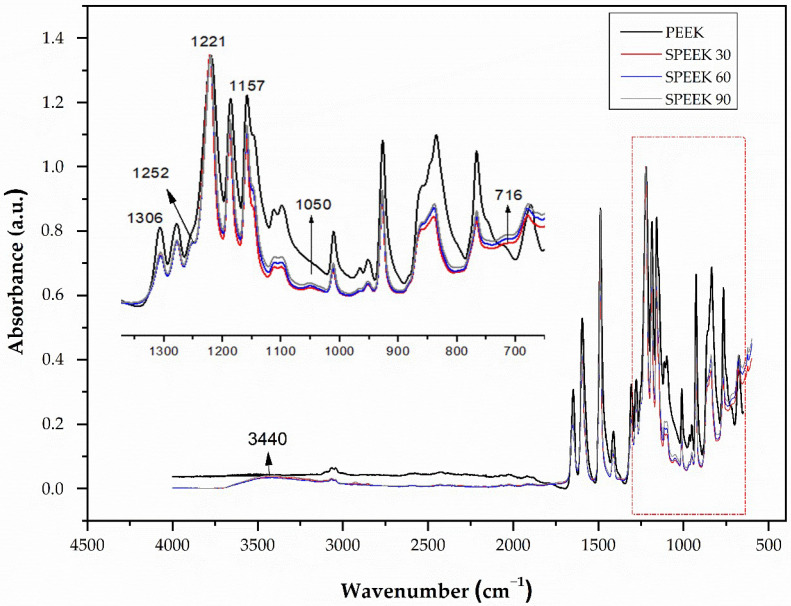
PEEK and SPEEK FTIR spectra with immersion times of 30, 60, and 90 s.

The FTIR spectra of unmodified PEEK (PEEK) and modified PEEK with sulfuric acid for 30, 60, and 90 s periods (SPEEK 30, SPEEK 60, and SPEEK 90) are shown in Figure 2.

For the PEEK spectrum, all characteristic bands are present. The most intense band is 1221 cm^−1^, referring to the asymmetric stretching of the C–O aromatic ether structure [29]. Bands at 1650, 1490, 926, 1157, and 1185 cm^−1^ correspond to the diphenylketone linkage [1]. The band present at 1306 cm^−1^ is associated with the C=O group of the ketone linkage, while the one at 1278 cm^−1^ is related to the resonance of the di-phenyl ether group. The C=O stretch corresponding to the benzophenone units is situated at 1594 and 1648 cm^−1^ [21]. These vibration profiles are typical of the PEEK polymer, and similar results have been reported in the literature [30].

The main structures of SPEEK and PEEK are similar. However, it is possible to identify differences in all investigated SPEEK samples. The bands related to the SPEEK spectrum present sulfonic acid groups at 3440, 1252, 1050, and 716 cm^−1^ [20]. The wideband at 3440 cm^−1^ belongs to the –OH vibration of the sulfonic acid functional group (SO_3_H) (occurring exclusively in the oxy-1,4-henyleneoxy rings) [RS(=O)_2_–OH] [17]. The vibration at 1050 cm^−1^ corresponds to the symmetrical stretching of the S=O bond, and the vibration at 1252 cm^−1^ is the asymmetric stretching of O=S=O [21]. The vibration at 716 cm^−1^ corresponds to the symmetrical stretching of the S–O bond [31,32]. It was also observed that the intensity of these bands increased with increasing sulfonation time.

**Figure 3:**
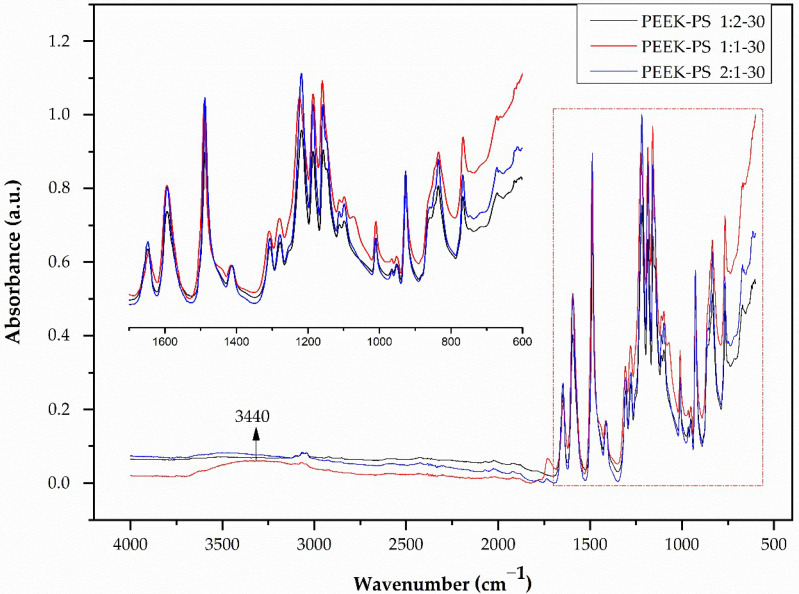
PEEK-PS FTIR spectra at concentrations of 1:2, 1:1, and 2:1 with 30 s of immersion in the piranha solution.

For the spectra presented for PEEK-PS with 30 s of immersion (Figure 3), a band at 3440 cm^−1^ was attributed to hydroxyl –OH vibration [17,33]. The appearance of this band indicates the oxidation of PEEK, which occurs through the oxygen released during the reaction of the hydrogen peroxide with sulfuric acid reacting directly with the aromatic ring of the benzene group.

For this case, there was probably no effect of sulfonation on the S=O band with absorption at 1050 cm^−1^ and at 1252 cm^−1^ (O=S=O), an effect reported by other authors [18,21]. This result probably can be explained because 30 s of sulfonation was not enough to allow the S–O group to be attached to the ketone radical. All other bands produced a similar absorption pattern.

**Figure 4:**
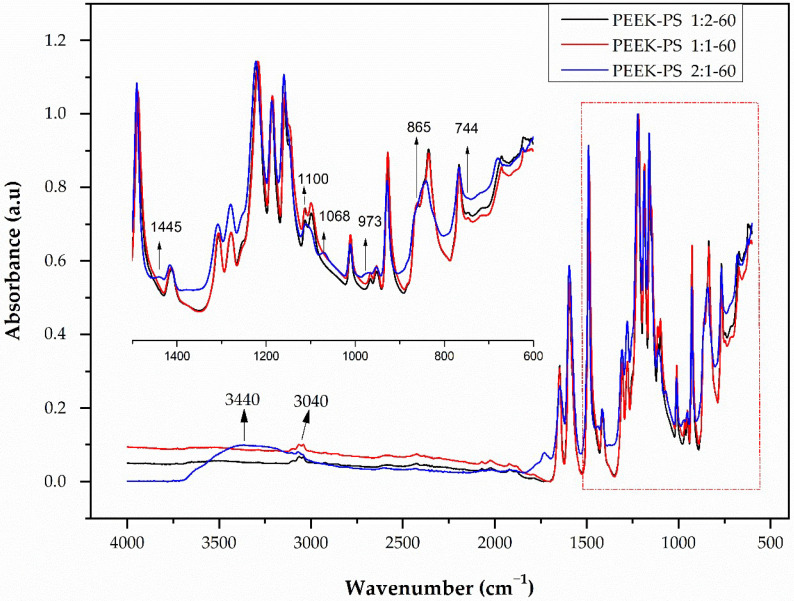
PEEK-PS FTIR spectra at concentrations of 1:2, 1:1, and 2:1 with a 60 s immersion time in the piranha solution.

In the PEEK-PS spectra for 60 s of immersion time (Figure 4), it was observed that the increase in the reaction time and concentration of the piranha solution contributed to the reaction kinetics. For PEEK-PS 2:1-60 an increase in band intensity occurred at 3440 cm^−1^, corresponding to the hydroxyl group (–OH) of the sulfonic acid functional group (SO_3_H). At this same concentration, the band relative to S=O at 1068 cm^−1^ presented a displacement, which indicates that this band can be shifted by 10 to 20 cm^−1^ to lower frequency by the conjugation effect. Schmidlin et al. [34] reported that when the surface of the PEEK was treated exclusively with sulfuric acid only the ether and carbonyl groups present between the aromatic rings were attacked. This fact may be due to the increase in the concentration of sulfuric acid that causes the SO_3_^−^ present in sulfuric acid and in the piranha solution to be responsible for the appearance of this band.

In the case of the piranha solution, the oxygen released during the reaction of the hydrogen peroxide with sulfuric acid reacts directly with the aromatic ring of the benzene group. This process leads to oxidation of the PEEK polymer, an increase in surface polarity, and aromatic ring opening, resulting in a greater quantity of functional groups available to bind to surrounding tissues [5,22].

The disappearance of the vibration bands of the CH=CH bond at 744 cm^−1^ and 865 cm^−1^, the reduction in the CH band at 3040 cm^−1^, and the appearance of bands at 1445 cm^−1^ corresponding to the angular deformation of –(CH_2_) out of the plane were observed [17]. These results suggest the opening of the aromatic ring of PEEK [5,22]. An increase in band intensity at 1734 cm^−1^, corresponding to the C=O ketone group was also detected. At 1100 cm^−1^, two bands that were related to C–O aliphatic ether became one. For the band at 973 cm^−1^, corresponding to the CH=CH vibration, a displacement occurred in addition to the decrease in intensity. The spectra corresponding to the concentrations of 1:2 and 1:1 showed no apparent change.

**Figure 5:**
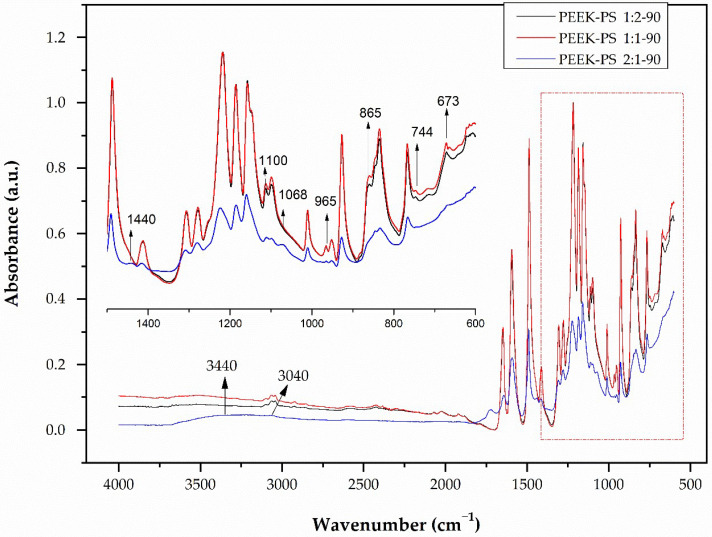
PEEK-PS FTIR spectra at concentrations of 1:2, 1:1, and 2:1 with 90 s of immersion in the piranha solution.

The spectra corresponding to the immersion time of 90 s in the 2:1 concentration (PEEK-PS 2:1-90), shown in Figure 5, showed even more intense band variations. The disappearance of the 865, 744, and 3040 cm^−1^ vibration bands and the reduction in the band intensity at 965 cm^−1^ (associated with the CH=CH bond) were also noticeable. The band at 673 cm^−1^, related to the angular deformation of the aromatic ring, also disappeared. This observation reinforces the possibility of the breaking of the aromatic ring as a function of the increase in the concentration and the time of reaction.

**Figure 6:**
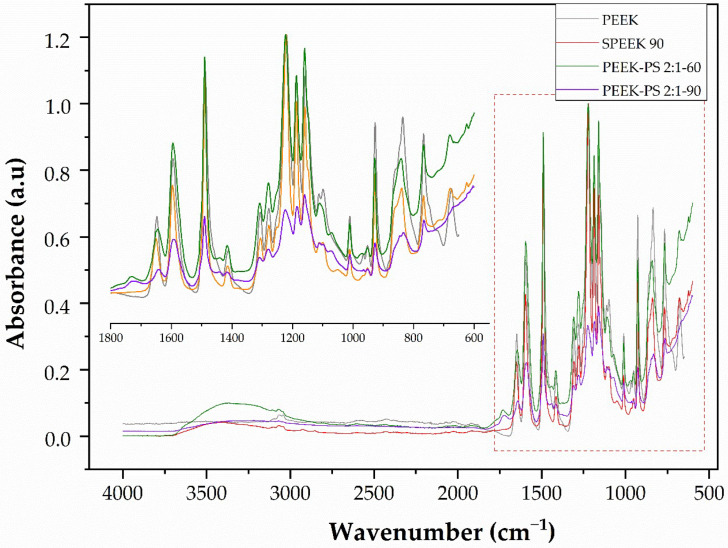
Comparative FTIR spectra of PEEK, SPEEK 90, PEEK-PS 2:1-60, and PEEK-PS 2:1-90.

**Figure 7:**
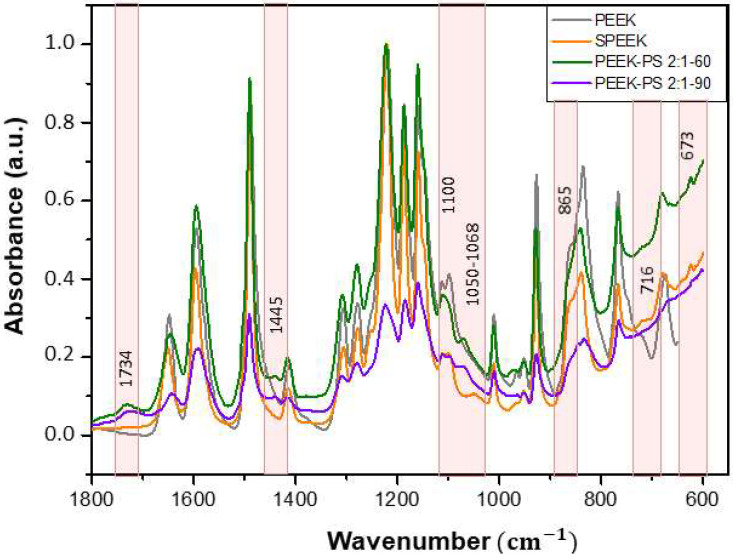
Comparative FTIR spectra of PEEK, SPEEK 90, PEEK-PS 2:1-60, and PEEK-PS 2:1-90, showing an enlargement of the region of 1800 to 550 cm^−1^.

Based on the observed results, it is possible to indicate the possible variations in the concentrations and times for further study. Regarding the treatment exclusively with sulfuric acid, only the 90 s immersion time was selected due to having the smallest increase in the intensity of the characteristic bands of the sulfonation effect. For the piranha solution treatment, the concentrations of 1:2 and 1:1 as well as the time of 30 s were not enough to cause the surface chemical modification. Therefore, at the concentration of 2:1, both the 60 and 90 s times showed better results and were selected to continue the investigation. The following spectra (Figure 6 and Figure 7) present a comparison between PEEK and the chosen treatments.

In Figure 7, the region from 1800 to 550 cm^−1^ is enlarged to provide a more detailed analysis of the differences between PEEK and the executed treatments.

During the oxidation reaction of the fresh mixture, various oxidants (O^+^, H_3_O^+^, OH^−^, HSO_4_^−^, and H_2_SO_5_) are formed transiently in a short time and result in H_2_SO_5_ (Caro’s acid) and H_2_O in a steady state with a low pH value. The chemical reaction rate increases exponentially at higher temperatures, as suggested by particle collision theory and transition state theory [35].

In the reaction of the piranha solution, Caro’s acid is originated, which is unstable and decomposes directly to form hydroxyl radicals, which are responsible for its oxidizing power [35,36]. The formation of the chemical oxidant is given in the reaction presented in Equation (1).

(1) $$\begin{array}{c} H_{2}{\mathrm{SO}}_{4}+H_{2}O_{2}↔H_{2}{\mathrm{SO}}_{5}+H_{2}O\\ \begin{array}{ccc} \;\;\;\;\;\;\;\;\;\;\;\;\;\;↑↓ & ↑↓ & ↑↓\\ \;\;\;\;\;\;\;\;\;\;\;\;\;\;2{\mathrm{HO}}^{-} & {\mathrm{HSO}}_{4} & +{\mathrm{HO}}^{-}. \end{array} \end{array}$$

From this perspective, it is possible to suggest that these hydroxyl radicals are responsible for the higher chemical modification on the PEEK surface (even if using pure sulfuric acid), a factor observed in the 3440 cm^−1^ band corresponding to the –OH bond. 

Therefore, with the IR results it was possible to select SPEEK 90, PEEK-PS 2:1-60, and PEEK-PS 2:1-90 as the samples with major potential to induce bioactivity. This choice was based on the fact that higher functionalization induces the presence of more functional groups that, in turn, promote bioactivity [14].

### 3.2. Student’s T-Test Applied to FTIR

The sulfonation of PEEK caused by the chemical modification was observed in PEEK-PS 2:1-60, PEEK-PS 2:1-90, and SPEEK 90. The t-test was applied to evaluate and compare if the treatments show significant differences in terms of the intensity of the bands that appeared for O–H and S=O. For each case, three measurements for the intensity were performed, followed by the t-test, which was utilized through the *p*-value in the averages corresponding to these measurements (Table 2).

The *p*-values determined for both cases were *p* < 0.05, so all the PEEK-PS 2:1-60 and PEEK-PS 2:1-90 vibration bands presented statistically significant differences when compared with SPEEK 90. Therefore, although the sulfonation effect is present in both treatments, the behavior was different concerning the intensity of the bands.

### 3.3. Scanning Electron Microscopy (SEM)

**Figure 8:**
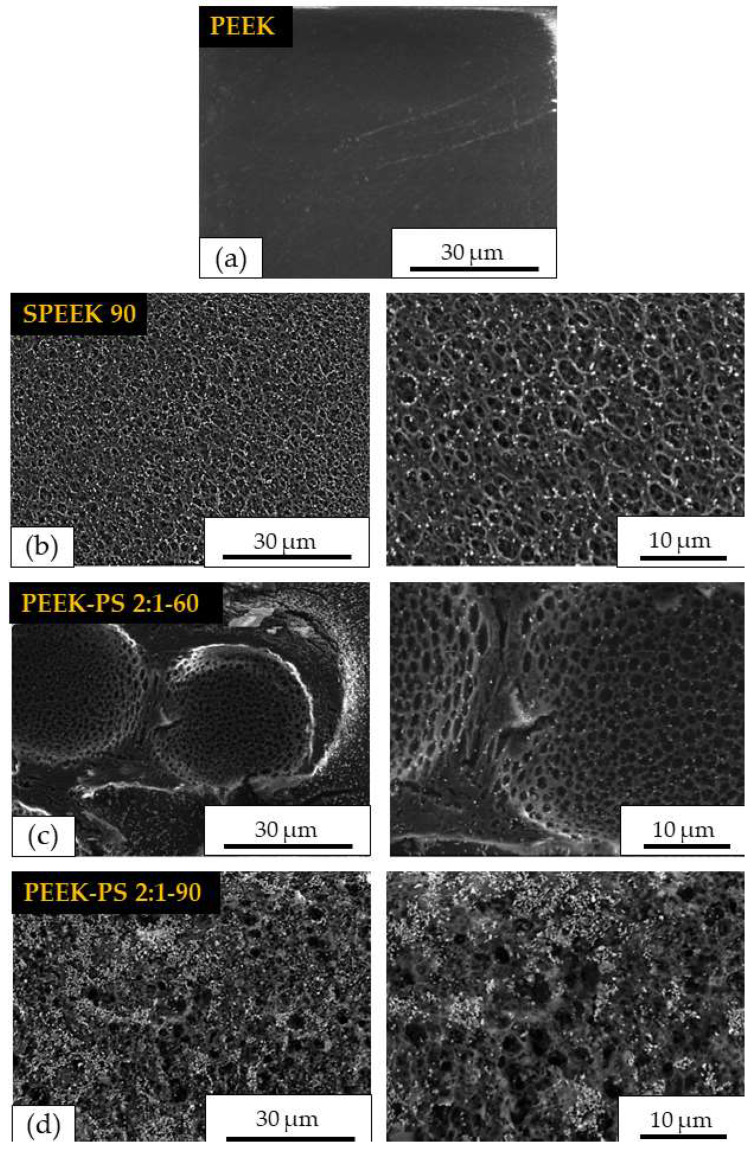
Comparison between surface micrographs of PEEK (**a**), SPEEK 90 (**b**), PEEK-PS 2:1-60 (**c**), and PEEK-PS 2:1-90 (**d**) with different magnifications.

In Figure 8, it is possible to observe a comparison between the morphology on the untreated PEEK surface, SPEEK 90 chemically modified only with sulfuric acid, and PEEK-PS 2:1 with 60 and 90 s of immersion in piranha solution. The micrographs corresponding to SPEEK, PEEK-PS 2:1-60, and PEEK-PS 2:1-90 demonstrate that the different treatments caused surface changes providing distinct characteristics in PEEK.

Regarding the micrograph corresponding to SPEEK 90, a porous surface with a uniform, homogeneous, and relatively regular morphology was observed (Figure 8b).

The pores observed on the SPEEK 90 surface (Figure 8b) have three-dimensional structures across the surface, are interconnected, and are relatively defined, with thin walls interconnecting one pore to another. In addition, they have varied diameters on the order of micrometers (<10 μm). Similar results were found in other studies [18,33,37].

Although the immersion time was lower than what has been reported in the literature [21], the proposed study was efficient, considering that the results found here confirmed the effect of surface sulfonation.

The sulfonation of PEEK occurs through polymer immersion in concentrated sulfuric acid at room temperature [37]. Subsequent to this step into the sulfuric acid, the SPEEK layer volume increases, and a small amount of H_2_SO_4_ remains on the surface. After immersion in distilled water, the SPEEK volume begins to decrease and passes to a solidified state. During the process, excess sulfuric acid penetrates the SPEEK and diffuses outward and thus many pores are formed during surface solidification, giving the sulfonated PEEK, or SPEEK. In addition, the chemical introduction of SO_3_H groups into the PEEK chain destroys the original compact structure and promotes pore formation. The porous structure of SPEEK presents characteristics of a three-dimensional network, which may be related to the hydrophilicity of SO_3_H groups [17]. During water immersion, the hydrophilic SO_3_H groups cause a continuous increase in the polymer chain volume, and consequently, the three-dimensional porous network is formed after the sulfuric acid remotion [21].

In the micrographs referring to the surface of PEEK-PS 2:1-60 and PEEK-PS 2:1-90 (Figure 8c, d), it was possible to observe that, although the difference in immersion time was not so high, the micrographs for the respective treatments presented different morphologies.

However, for the treatment with PEEK-PS 2:1-60, the surface presented two distinct regions, one more densified and irregular, with few pores, grooves, and cracks (Figure 8c) and another with the presence of large deep cavities with uniform micrometric internal pores interconnected to each other through thin walls.

In the PEEK-PS 2:1-90, it was observed that this treatment promoted a pronounced change on the surface (Figure 8d) when compared to the others. This aspect was expected previously, considering that this solution acts as a potent corrosive and oxidant agent [26], which hydroxylates the surfaces and makes them hydrophilic, thus improving their adhesive and microroughness properties [24,38].

In addition, the surface exhibited greater heterogeneity in the pores, presenting several sizes on the order of micrometers, thus creating a more open structure [36].

The representative micrographs of the PEEK surface treated with the piranha solution did not resemble what has been reported in the literature. This can be explained by the different proportions of sulfuric acid and hydrogen peroxide and the immersion times used in this study.

Authors reported no significant changes using piranha solution [23,26,33,39]. However, this study registered some alterations reflecting ideal conditions of immersion associated with stoichiometrically calculated proportions, which provided the chemical modification.

The changes caused by both functionalization methods, piranha solution and sulfuric acid, were efficient in modifying the PEEK surface. The piranha solution method promoted more pronounced changes on the surface, especially the PEEK-PS 2:1-90 sample. The formation of three-dimensional networks with interconnected pores is one of the factors that has a direct effect on cell adhesion [40].

### 3.4. Surface Roughness Measurement

**Figure 9:**
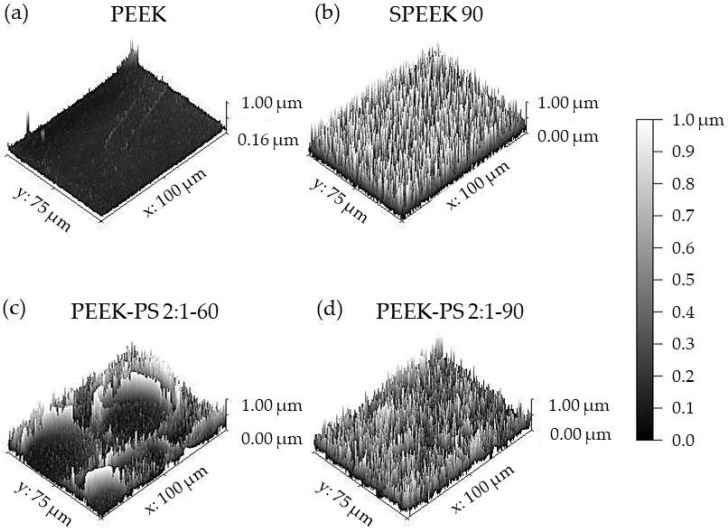
Comparison between topographic images of PEEK (**a**), SPEEK 90 (**b**), PEEK-PS 2:1-60 (**c**), and PEEK-PS 2:1-90 (**d**) obtained from SEM micrographs using Gwyddion software.

**Figure 10:**
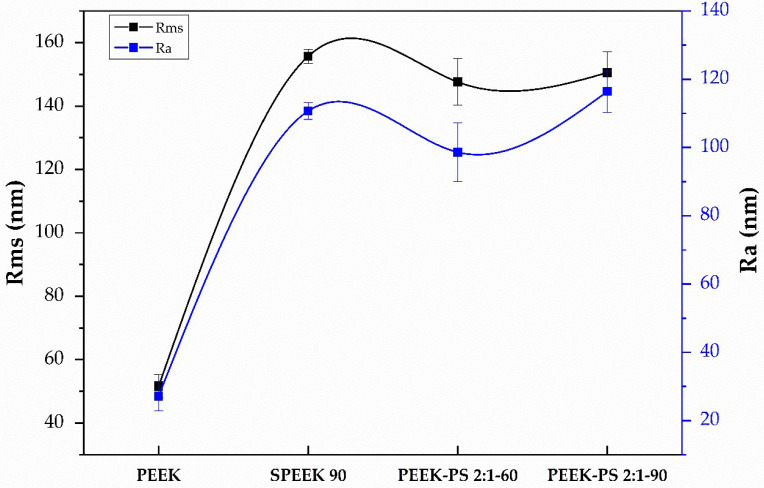
Surface roughness of untreated PEEK, SPEEK 90, and piranha-solution-treated PEEK-PS 2:1-60 and PEEK-PS 2:1-90 obtained from SEM micrographs using Gwyddion software. Results are presented as means ± SD (*n* = 3). *p*-value < 0.05, *p* = value > 0.05.

Implant surface roughness and topography are important parameters that influence adsorption, adhesion, migration, and cell differentiation. Moreover, the substrate topography presents a straight effect on the ability of cells to produce organized arrays [40]. The topography images of PEEK, SPEEK 90, PEEK-PS 2:1-60, and PEEK-PS 2:1-90, estimated through surface roughness, were obtained from SEM micrographs using the Gwyddion 2.45 software. Topography analyses represented by differences in surface characteristics are presented in Figure 9. Root mean square (Rms) and the mean roughness (roughness average, Ra) data are evaluated in Figure 10.

According to the results, the untreated PEEK sample (Figure 9a) had minimal surface roughness due to the lack of chemical surface treatment. PEEK-PS 2:1-60 (Figure 9c) showed irregular and heterogeneous topography compared to SPEEK 90 (Figure 9b) and PEEK-PS 2:1-90 (Figure 9d), which showed a homogeneous topography.

The functionalization effect of PEEK with sulfuric acid (SPEEK 90) is noticed as an increase in the Rms and Ra values compared to the PEEK samples. The Rms value increased from 52 ± 5 nm to 156 ± 3 nm compared to the untreated PEEK and H_2_SO_4_-functionalized samples (SPEEK 90). PEEK and SPEEK 90 presented mean roughness values of 27 ± 6 nm and 111 ± 3 nm, respectively (Figure 10).

The treatment with piranha solution exhibited an increase in the Rms and Ra values directly proportional to the immersion time. The Rms and Ra values in the PEEK-PS 2:1-60 and PEEK-PS 2:1-90 samples changed from 148 ± 9 nm to 151 ± 8 nm and from 99 ± 11 nm to 116 ± 7 nm, respectively. The increase in piranha solution immersion time from 60 to 90 s did not significantly affect the Rms and Ra parameters.

Rms and Ra data were also evaluated with a t-test applied in untreated PEEK samples and samples treated with H_2_SO_4_ and piranha solution. It was observed that the changes caused by chemical treatments statistically affected the PEEK roughness (*p*-value < 0.05). However, there were no statistically significant differences between the treatments (*p*-value > 0.05).

The surface roughness results are in agreement with the literature. The effect of piranha solution treatment on polyvinylidene fluoride (PVDF) roughness was evaluated by Al-Gharabli et al. [36]. Their results presented increases in Rms values in PVDF samples functionalized with piranha solution. Other works also reported an increment in PEEK surface roughness related to the use of sulfuric acid as a surface treatment and increases in the immersion time of the samples [18]. Jurak, Wiącek, and Terpiłowski [13] reported an increase in roughness values for the PEEK surface caused by plasma modification associated with surface coating, especially for the treatment with argon plasma, which obtained Ra = 2.29 μm.

In summary, the use of sulfuric acid and piranha solution significantly modified the roughness of untreated PEEK. The SPEEK 90 and PEEK-PS 2:1-90 samples showed higher Rms and RA values without significant differences. A rougher surface can affect cell adhesion and differentiation [2,41]. In addition, the greater surface roughness of the material generally leads to better adhesion and growth of bone cells [42].

### 3.5. Contact Angle

**Figure 11:**
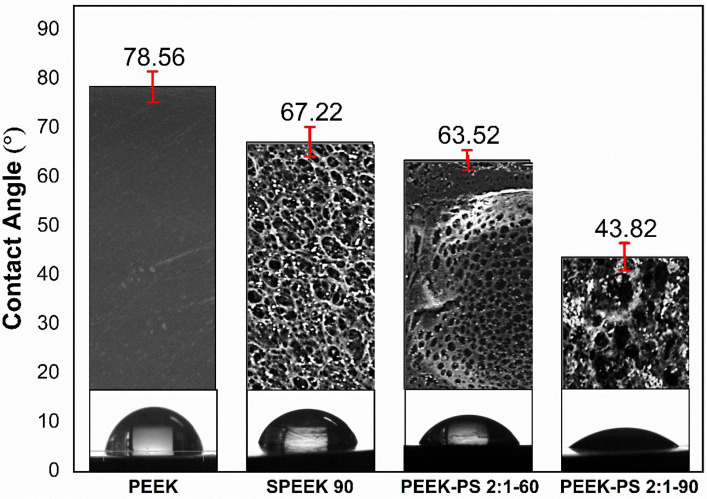
Surface contact angle measurements through the contact angle of the water on untreated PEEK, SPEEK 90, and piranha-solution-treated PEEK-PS 2:1-60 and PEEK-PS 2:1-90 obtained from SEM micrographs using Gwyddion software. Results are presented as means ± SD (*n* = 5). *p*-value > 0.05.

The contact angle is an important parameter that relates wettability to the interactions between the functional groups on the material surface and the tissue adjacent to the implant [10]. Figure 11 shows the contact angle measurements of PEEK with and without the treatments. For all samples, the chemical modification on the PEEK surface improved the wettability.

PEEK exhibited a contact angle of 78.56°, followed by SPEEK 90 (67.22°), PEEK-PS 2:1-60 (63.52°), and PEEK-PS 2:1-90 (43.82°). PEEK-PS 2:1-90 demonstrated a more hydrophilic surface, with a reduction of 44% when compared to the untreated PEEK, similar to that reported in the literature [19,20]. The introduction of the hydrophilic HSO_3_^−^ functional group pointed by FTIR reflects the effect of the chemical modification on the PEEK surface, especially in the noticeable decrease in the PEEK-PS 2:1-90 sample contact angle, caused by the increase in the immersion time in the piranha solution.

Those results exhibited effectiveness similar to other methods of functionalization. For example, Han et al. [43] observed a reduction in the contact angle from 89.5 ±  2.5° (untreated PEEK) to 41.1  ±  7.3° and 38.0  ±  3.1° for PEEK treated with argon plasma and oxygen plasma, respectively. Other experiments involving the PEEK surface functionalization by plasma reduced the contact angle. The initial measurement of around 70° for the untreated PEEK decreased to 7° after modification with oxygen plasma. This effect occurs due to the chain scissions and the subsequent formation of polar groups as a consequence of their combination with O_2_ [11].

These observed effects of the chemical modifications are promising for decreasing the bioinert character of the PEEK surface [13,44]. Indeed, a surface with more pronounced hydrophilicity has been considered an important parameter to increase cell adhesion [45,46]. Consequently, it is possible to accelerate bone healing and implant osseointegration processes, as demonstrated by other studies that found an improvement in osseointegration capability and the in vivo increment of sulfonated PEEK samples [21,47].

Thus, the reduction in the contact angle, especially in the PEEK-PS 2:1-90 sample, is promising to decrease the bioinert character of the PEEK surface since a surface with high hydrophilicity is more favorable to increased interaction, proliferation, and cell adhesion, which can accelerate bone healing, osteogenic capacity, and the osseointegration of the implant [21,45,46,47,48].

### 3.6. In Vitro Bioactivity

**Figure 12:**
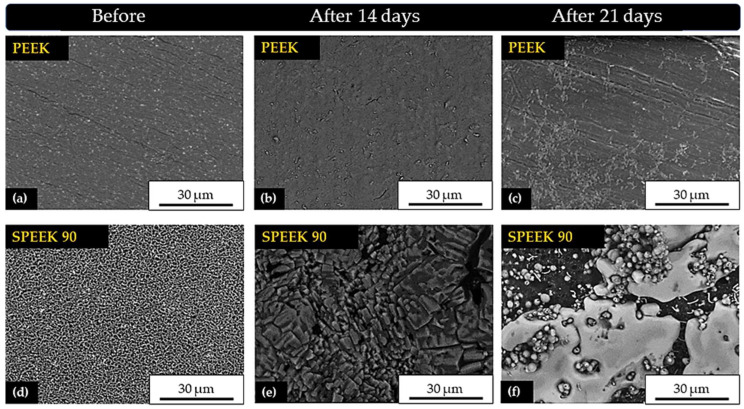
SEM micrographs of PEEK and SPEEK 90 surfaces, (**a**,**d**) and after immersion in SBF for 14 (**b**,**e**) and 21 days (**c**,**f**), with 3000× magnification.

Figure 12a–c show PEEK without treatment and after 14 and 21 days of immersion in PBS, respectively.

According to the results on the PEEK surface after 14 days (Figure 12b), there was no evidence of calcium phosphate precipitate formation, similar to what was observed by Ma and Guo [49]. However, at the end of 21 days (Figure 12c), it was possible to notice the formation of particles in some specific regions that may have formed in the sanding and polishing processes. Similar results were observed by Fook [50] when they applied mechanical polishing and sanding treatments on the surface of PEUAPM (ultra high molecular weight polyethylene) polymer.

As for the chemically treated samples, in SPEEK 90 there was a change in the surface morphology. In this case, after 14 days there was a preferential deposition of a monophasic calcium phosphate layer with heterogeneous geometry and different sizes along the surface (Figure 12e). Ren et al. [51] also confirmed the formation of a dense coating on the surface of modified PEEK after the first days of immersion in SBF.

Subsequently, after 21 days (Figure 12f) a biphasic layer was formed on this surface, with the preferable presence of elongated plates and spheres. In this case, these results may suggest the existence of apatite precipitates. Similar results indicated that this treatment promoted the bioactive capacity on the surface of PEEK [18,21,28,49,50,52].

Zhao et al. [21] observed that apatite formation is induced by sulfonation. The comprehension of this mechanism may involve the electrostatic interaction between the functional groups and the ions in SBF. In a dry environment, the net charge of the SO_3_H groups is supposed to be zero. In an aqueous medium, the SO_3_H group dissociates into SO_3_^−^ and H^+^, and the SPEEK surface after immersion in SBF becomes negatively charged. Previous studies showed that SBF can induce heterogeneous nucleation and apatite growth, considering its ionic nature, where electrostatic interaction triggers initial nucleation. Because of that, the positively charged calcium ions (Ca^2+^) in the SBF are first incorporated on the surfaces.

**Figure 13:**
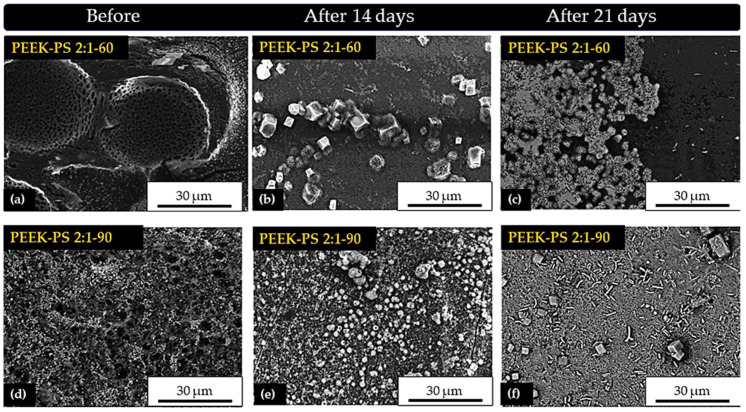
SEM micrographs of PEEK-PS 2:1-60 and PEEK-PS 2:1-90 surfaces, before (**a**,**d**) and after immersion in SBF for 14 (**b**,**e**) and 21 days (**c**,**f**), with 3000× magnification.

For PEEK-PS 2:1-60 after 14 days of immersion (Figure 13b), a calcium phosphate phase and some crystal deposits were formed, which could be related to NaCl. After 21 days (Figure 13c), there was the formation of another calcium phosphate phase arranged in an agglomerated form with the presence of spherical particles.

It should also be emphasized that the formation of a bioactive layer in PEEK-PS 2:1-60 increased with the immersion time: a behavior possibly directly related to the increase in the solution pH after immersion. Studies have shown that this increase plays a significant role in calcium phosphate layer formation, considering that it occurs due to ionic supersaturation in the SBF solution, promoting the precipitation of calcium and phosphorus ions on the surface [53].

Finally, on the surface of PEEK-PS 2:1-90 after 14 days (Figure 13e), there was a bioactive coating of particles with spherical and irregular shapes, in addition to the presence of agglomerates. At the end of 21 days (Figure 13f), this coating exhibited growth and larger densification over almost the entire surface. Therefore, it turned into a biphasic layer with agglomerates of needle-shaped particles.

In all applied treatments, the surfaces became bioactive, although the PEEK-PS 2:1-90 sample functionalized by piranha solution had a more pronounced response. These results are due to the SO_3_H groups that are the main reason for the increase in the capacity to form apatite. At the first moment after 14 days, for the three treatments, there was the formation of a preferential phase, and after the exchange of the SBF solution under an already covered surface, a new ion equilibrium occurred that induced a new phase formation above the previous one. Because of this, there was the formation of distinct phases at the end of 14 and 21 days. Lastly, the results show that both treatments were effective. They enabled the calcium deposition, probably due to the higher number of functional groups on the modified surface, thus promoting bioactivity and presenting a potential for application as a biomaterial.

## 4. Conclusions

This work aimed to achieve bioactivity on the PEEK surface using a chemical treatment of piranha solution, overcoming the surface modification by conventional sulfuric acid and reaching effectiveness analogous to the most used physical treatments for polymers. Under this point of view, different times and concentrations were tested, aiming for an optimal condition of functionalization. In the end, it is possible to propose the piranha solution with a shorter reaction time for the functionalization of the PEEK surface. The evaluated treatments were effective in the bioactivation of the surface of PEEK samples since this property was achieved using sulfuric acid and piranha solution with shorter functionalization times. Sulfonation was confirmed for all treatments. However, the piranha solution allowed the opening of the aromatic ring, providing greater availability of functional groups, which favors surface roughness and bioactivity. In both treatments, there was a change in the surface morphology. In statistical terms, the piranha solution with the highest concentration and longest immersion times promoted pronounced heterogeneity in the surface pores, affecting the roughness of the untreated PEEK. The treatments caused a reduction in the water contact angle, which were increased by using the piranha solution, making the surface more hydrophilic, which is desirable for biological interaction. Finally, both treatments allowed calcium deposition, probably due to the higher amount of available functional groups on the surface. However, the surface bioactivity of PEEK was observed more significantly after using the piranha solution, especially the PEEK PS 2:1-90 sample. Therefore, the use of piranha solution can be considered a promising alternative route for the functionalization of PEEK-based implants with the potential for application as a biomaterial.

## Figures and Tables

**Table 1:** Test parameters and sample identification.

Treatment	Immersion Time (s)	Codification
Sulfuric Acid H_2_SO_4_ (98%)	30 60 90	SPEEK 30 SPEEK 60 SPEEK 90
Piranha Solution 1:2 *v*/*v* H_2_SO_4_ (98%):H_2_O_2_ (35%)	30 60 90	PEEK-PS 1:2-30 PEEK-PS 1:2-60 PEEK-PS 1:2-90
Piranha Solution 1:1 *v*/*v* H_2_SO_4_ (98%):H_2_O_2_ (35%)	30 60 90	PEEK-PS 1:1-30 PEEK-PS 1:1-60 PEEK-PS 1:1-90
Piranha Solution 2:1 *v*/*v* H_2_SO_4_ (98%):H_2_O_2_ (35%)	30 60 90	PEEK-PS 2:1-30 PEEK-PS 2:1-60 PEEK-PS 2:1-90

**Table 2:** T-test applied to the vibration bands at O–H and S=O: comparison between the *p*-values of SPEEK 90 and PEEK-PS 2:1-60, and SPEEK 90 and PEEK-PS 2:1-90. All tests were performed in triplicate (*n* = 3). For *p*-value < 0.05, the result is considered significant, and is assumed that the sample means are not statistically equal. In the case of *p*-value > 0.05, the result is not considered significant, and the sample means are statistically the same.

Samples	Vibration Bands (O–H) *p*-Value	Vibration Bands (S=O) *p*-Value
(SPEEK 90 and PEEK-PS 2:1-60)	0.002768897	0.00074459
(SPEEK 90 and PEEK-PS 2:1-90)	0.04471751	0.002609884

## Sample Availability

Samples of the compounds are available from the authors.

## References

[1] Kurtz S.M. (2012). An overview of PEEK biomaterials. PEEK Biomaterials Handbook.

[2] Liu X., Gan K., Liu H., Song X., Chen T., Liu C. (2017). Antibacterial properties of nano-silver coated PEEK prepared through magnetron sputtering. Dent. Mater..

[3] Ma R., Tang T. (2014). Current strategies to improve the bioactivity of PEEK. Int. J. Mol. Sci..

[4] Deng L., Deng Y., Xie K. (2017). AgNPs-decorated 3D printed PEEK implant for infection control and bone repair. Colloids Surf. B Biointerfaces.

[5] Hallmann L., Mehl A., Sereno N., Hämmerle C.H. (2012). The improvement of adhesive properties of PEEK through different pre-treatments. Appl. Surf. Sci..

[6] Evans N.T., Torstrick F.B., Lee C.S., Dupont K.M., Safranski D.L., Chang W.A., Macedo A.E., Lin A.S., Boothby J.M., Whittingslow D.C. (2015). High-strength, surface-porous polyether-ether-ketone for load-bearing orthopedic implants. Acta Biomater..

[7] Durham III J.W., Rabiei A. (2016). Deposition, heat treatment and characterization of two layer bioactive coatings on cylindrical PEEK. Surf. Coat. Technol..

[8] Najeeb S., Zafar M.S., Khurshid Z., Siddiqui F. (2016). Applications of polyetheretherketone (PEEK) in oral implantology and prosthodontics. J. Prosthodont. Res..

[9] Wiącek A.E., Terpiłowski K., Jurak M., Worzakowska M. (2016). Effect of low-temperature plasma on chitosan-coated PEEK polymer characteristics. Eur. Polym. J..

[10] Yuan B., Cheng Q., Zhao R., Zhu X., Yang X., Yang X., Zhang K., Song Y., Zhang X. (2018). Comparison of osteointegration property between PEKK and PEEK: Effects of surface structure and chemistry. Biomaterials.

[11] Sundriyal P., Sahu M., Prakash O., Bhattacharya S. (2021). Long-term surface modification of PEEK polymer using plasma and PEG silane treatment. Surf. Interfaces.

[12] Kyzioł A., Kyzioł K. (2018). Surface functionalization with biopolymers via plasma-assisted surface grafting and plasma-induced graft polymerization—materials for biomedical applications. Biopolymer Grafting.

[13] Jurak M., Wiącek A.E., Terpiłowski K. (2016). Properties of PEEK-supported films of biological substances prepared by the Langmuir-Blodgett technique. Colloids Surf. A Physicochem. Eng. Asp..

[14] Bose S., Robertson S.F., Bandyopadhyay A. (2018). Surface modification of biomaterials and biomedical devices using additive manufacturing. Acta Biomater..

[15] Jin X., Bishop M.T., Ellis T.S., Karasz F.E. (1985). A sulphonated poly (aryl ether ketone). Br. Polym. J..

[16] Bailly C., Williams D.J., Karasz F.E., MacKnight W.J. (1987). The sodium salts of sulphonated poly (aryl-ether-ether-ketone)(PEEK): Preparation and characterization. Polymer.

[17] Zaidi S.J. (2003). Polymer sulfonation-A versatile route to prepare proton-conducting membrane material for advanced technologies. Arab. J. Sci. Eng..

[18] Almasi D., Izman S., Assadian M., Ghanbari M., Kadir M.A. (2014). Crystalline HA coating on PEEK via chemical deposition. Appl. Surf. Sci..

[19] Wang W., Luo C., Huang J., Edirisinghe M. (2019). PEEK surface modification by fast ambient-temperature sulfonation for bone implant applications. J. R. Soc. Interface.

[20] Montero J.F., Tajiri H.A., Barra G.M., Fredel M.C., Benfatti C.A., Magini R.S., Pimenta A.L., Souza J.C. (2017). Biofilm behavior on sulfonated poly (ether-ether-ketone) (sPEEK). Mater. Sci. Eng. C.

[21] Zhao Y., Wong H.M., Wang W., Li P., Xu Z., Chong E.Y., Yan C.H., Yeung K.W., Chu P.K. (2013). Cytocompatibility, osseointegration, and bioactivity of three-dimensional porous and nanostructured network on polyetheretherketone. Biomaterials.

[22] Stawarczyk B., Jordan P., Schmidlin P.R., Roos M., Eichberger M., Gernet W., Keul C. (2014). PEEK surface treatment effects on tensile bond strength to veneering resins. J. Prosthet. Dent..

[23] Uhrenbacher J., Schmidlin P.R., Keul C., Eichberger M., Roos M., Gernet W., Stawarczyk B. (2014). The effect of surface modification on the retention strength of polyetheretherketone crowns adhesively bonded to dentin abutments. J. Prosthet. Dent..

[24] Zandparsa R., Talua N.A., Finkelman M.D., Schaus S.E. (2014). An in vitro comparison of shear bond strength of zirconia to enamel using different surface treatments. J. Prosthodont..

[25] Melo R., Souza R., Dursun E., Monteiro E., Valandro L., Bottino M. (2015). Surface treatments of zirconia to enhance bonding durability. Oper. Dent..

[26] Hallmann L., Ulmer P., Lehmann F., Wille S., Polonskyi O., Johannes M., Köbel S., Trottenberg T., Bornholdt S., Haase F. (2016). Effect of surface modifications on the bond strength of zirconia ceramic with resin cement resin. Dent. Mater..

[27] Eulálio H.Y.C., Vieira M., Fideles T.B., Tomás H., Silva S.M., Peniche C.A., Fook M.V.L. (2020). Physicochemical Properties and Cell Viability of Shrimp Chitosan Films as Affected by Film Casting Solvents. I-Potential Use as Wound Dressing. Materials.

[28] Kokubo T., Takadama H. (2006). How useful is SBF in predicting in vivo bone bioactivity?. Biomaterials.

[29] Nakamura H., Nakamura T., Noguchi T., Imagawa K. (2006). Photodegradation of PEEK sheets under tensile stress. Polym. Degrad. Stab..

[30] Patel K., Doyle C.S., James B.J., Hyland M.M. (2010). Valence band XPS and FT-IR evaluation of thermal degradation of HVAF thermally sprayed PEEK coatings. Polym. Degrad. Stab..

[31] Doğan H., Inan T.Y., Koral M., Kaya M. (2011). Organo-montmorillonites and sulfonated PEEK nanocomposite membranes for fuel cell applications. Appl. Clay Sci..

[32] Dutra Filho J.C., Santos T.R.d., Gomes A.d.S. (2014). Nanostructured Polyelectrolytes Based on SPEEK/TiO_2_ for Direct Ethanol Fuel Cells (DEFCs). Polímeros.

[33] Silthampitag P., Chaijareenont P., Tattakorn K., Banjongprasert C., Takahashi H., Arksornnukit M. (2016). Effect of surface pretreatments on resin composite bonding to PEEK. Dent. Mater. J..

[34] Schmidlin P.R., Stawarczyk B., Wieland M., Attin T., Hämmerle C.H., Fischer J. (2010). Effect of different surface pre-treatments and luting materials on shear bond strength to PEEK. Dent. Mater..

[35] Chu M.M., Chou J.-H. (2012). Fluid-kinetics enhanced selective etching process of NiPt film by piranha chemistry in silicide formation for complementary metal oxide semiconductor fabrication. Thin Solid Film..

[36] Al-Gharabli S., Kujawa J., Mavukkandy M.O., Arafat H.A. (2017). Functional groups docking on PVDF membranes: Novel Piranha approach. Eur. Polym. J..

[37] Ouyang L., Zhao Y., Jin G., Lu T., Li J., Qiao Y., Ning C., Zhang X., Chu P.K., Liu X. (2016). Influence of sulfur content on bone formation and antibacterial ability of sulfonated PEEK. Biomaterials.

[38] Lohbauer U., Zipperle M., Rischka K., Petschelt A., Müller F.A. (2008). Hydroxylation of dental zirconia surfaces: Characterization and bonding potential. J. Biomed. Mater. Res. Part B: Appl. Biomater. Off. J. Soc. Biomater. Jpn. Soc. Biomater. Aust. Soc. Biomater. Korean Soc. Biomater..

[39] Siddiq A.R., Kennedy A.R. (2015). Porous poly-ether ether ketone (PEEK) manufactured by a novel powder route using near-spherical salt bead porogens: Characterisation and mechanical properties. Mater. Sci. Eng. C.

[40] Su Y., Luo C., Zhang Z., Hermawan H., Zhu D., Huang J., Liang Y., Li G., Ren L. (2018). Bioinspired surface functionalization of metallic biomaterials. J. Mech. Behav. Biomed. Mater..

[41] Yamashita D., Machigashira M., Miyamoto M., Takeuchi H., Noguchi K., Izumi Y., Ban S. (2009). Effect of surface roughness on initial responses of osteoblast-like cells on two types of zirconia. Dent. Mater. J..

[42] Gan K., Liu H., Jiang L., Liu X., Song X., Niu D., Chen T., Liu C. (2016). Bioactivity and antibacterial effect of nitrogen plasma immersion ion implantation on polyetheretherketone. Dent. Mater..

[43] Han X., Sharma N., Spintzyk S., Zhou Y., Xu Z., Thieringer F.M., Rupp F. (2022). Tailoring the biologic responses of 3D printed PEEK medical implants by plasma functionalization. Dent. Mater..

[44] Jurak M., Wiącek A.E., Ładniak A., Przykaza K., Szafran K. (2021). What affects the biocompatibility of polymers?. Adv. Colloid Interface Sci..

[45] Zhu Y., Cao Z., Peng Y., Hu L., Guney T., Tang B. (2019). Facile Surface Modification Method for Synergistically Enhancing the Biocompatibility and Bioactivity of Poly(ether ether ketone) That Induced Osteodifferentiation. ACS Appl. Mater. Interfaces.

[46] Gittens R.A., Scheideler L., Rupp F., Hyzy S.L., Geis-Gerstorfer J., Schwartz Z., Boyan B.D. (2014). A review on the wettability of dental implant surfaces II: Biological and clinical aspects. Acta Biomater..

[47] Hieda A., Uemura N., Hashimoto Y., Toda I., Baba S. (2017). In vivo bioactivity of porous polyetheretherketone with a foamed surface. Dent. Mater. J..

[48] Rupp F., Gittens R.A., Scheideler L., Marmur A., Boyan B.D., Schwartz Z., Geis-Gerstorfer J. (2014). A review on the wettability of dental implant surfaces I: Theoretical and experimental aspects. Acta Biomater..

[49] Ma R., Guo D. (2019). Evaluating the bioactivity of a hydroxyapatite-incorporated polyetheretherketone biocomposite. J. Orthop. Surg. Res..

[50] Fook M.V.L. (2005). Desenvolvimento de técnica de deposição de hidroxiapatita pelo método biomimético na superfície polietileno de ultra-alto peso molecular para aplicação como biomaterial. Tese, Doutorado em Química.

[51] Ren Y., Sikder P., Lin B., Bhaduri S.B. (2018). Microwave assisted coating of bioactive amorphous magnesium phosphate (AMP) on polyetheretherketone (PEEK). Mater. Sci. Eng. C.

[52] Hong W., Guo F., Chen J., Wang X., Zhao X., Xiao P. (2018). Bioactive glass–chitosan composite coatings on PEEK: Effects of surface wettability and roughness on the interfacial fracture resistance and in vitro cell response. Appl. Surf. Sci..

[53] Tulyaganov D.U., Makhkamov M.E., Urazbaev A., Goel A., Ferreira J.M. (2013). Synthesis, processing and characterization of a bioactive glass composition for bone regeneration. Ceram. Int..

